# Antibody-CD20-interferon-alpha fusion protein has superior in vivo activity against human B cell lymphomas compared to Rituximab, and enhanced complement-dependent cytotoxicity in vitro

**DOI:** 10.1186/2051-1426-1-S1-P263

**Published:** 2013-11-07

**Authors:** Reiko Yamada, Kristopher Steward, Gataree Ngarmchamnanrith, Ryan Trinh, Sanjay Khare, Raj Sachdev, Iqbal Grewal, Sherie Morrison, John Timmerman

**Affiliations:** 1 Medicine - Hematology/Oncology, UCLA, Los Angeles, CA, USA; 2 Microbiology, Immunology, and Molecular Genetics, UCLA, Los Angeles, CA, USA; 3 ImmunGene, Inc. and Valor Biotherapeutics, Thousand Oaks, CA, USA

## Background

We previously reported an anti-CD20-interferon-alpha (IFNα) fusion protein able to induce apoptosis and promote in vivo eradication of a human CD20-expressing mouse B cell lymphoma [Xuan et al, Blood 2010]. We now report the activity of a recombinant anti-CD20-human IFNα fusion protein against human non-Hodgkin B cell lymphomas (NHL).

## Methods

Anti-CD20-hIFNα was evaluated against a panel of human Burkitt, diffuse large B cell (DLBCL), and mantle cell lymphoma cell lines. Proliferation was measured by [3H]-thymidine, complement-dependent cytotoxicity (CDC) by PI flow cytometry, and antibody-dependent cellular cytotoxicity (ADCC) by LDH release using PBMC effectors. NHL xenografts were grown in SCID mice.

## Results

Anti-CD20-hIFNα induced stronger growth inhibition than rituximab, particularly against Burkitt and germinal center-type DLBCL NHLs. Tumor growth inhibition by anti-CD20-hIFNα was associated with substantial apoptosis in some cell lines. Anti-CD20-hIFNα exhibited potent ADCC activity against Daudi, Ramos, and Raji cells, identical to rituximab. Surprisingly, anti-CD20-hIFNα exhibited superior CDC compared to rituximab against Daudi, Ramos, and Raji cells that was dependent upon linkage of IFNα to the anti-CD20 antibody, and correlated with improved complement fixation. Importantly, against Raji NHL xenograft tumors in SCID mice, anti-CD20-hIFNα achieved superior efficacy compared to rituximab (p=0.0015) and control fusion protein (p<0.0001). At antibody doses at which Raji xenograft tumors progressed through rituximab, anti-CD20-hIFNα eradicated 50-88% of established tumors. Non-targeted control fusion protein had only minor effects on tumor growth.

## Conclusions

Anti-CD20-hIFNα has stronger direct anti-proliferative and CDC activities than rituximab against human NHL while retaining potent ADCC activity, and also has the ability to eradicate established NHL xenografts in vivo. These results support the further development of anti-CD20-hIFNα for the treatment of B cell NHL, and a phase I first-in-human clinical trial is currently being planned.

